# Using Mobile Applications to Increase Physical Activity: A Systematic Review

**DOI:** 10.3390/ijerph17218238

**Published:** 2020-11-07

**Authors:** Laura Pradal-Cano, Carolina Lozano-Ruiz, José Juan Pereyra-Rodríguez, Francesc Saigí-Rubió, Anna Bach-Faig, Laura Esquius, F. Xavier Medina, Alicia Aguilar-Martínez

**Affiliations:** 1 Information Systems, Hospital Clínic de Barcelona, 08036 Barcelona, Spain; laurapradal@gmail.com; 2 Faculty of Health Sciences, Universitat Oberta de Catalunya (UOC), 08018 Barcelona, Spain; clozano01@uoc.edu; 3 UGC Dermatology, University Hospital Virgen del Rocío, 41013 Sevilla, Spain; pe3reyra@gmail.com; 4 Interdisciplinary Research Group on ICTs, Faculty of Health Sciences, Universitat Oberta de Catalunya (UOC), 08018 Barcelona, Spain; fsaigi@uoc.edu; 5 FoodLab Research Group (2017SGR 83), Faculty of Health Sciences, Universitat Oberta de Catalunya (UOC), 08018 Barcelona, Spain; abachf@uoc.edu (A.B.-F.); lesquius@uoc.edu (L.E.); fxmedina@uoc.edu (F.X.M.); 6 Food and Nutrition Area, Barcelona Official College of Pharmacists, 08009 Barcelona, Spain; 7 School of Psychology, Education and Sport Sciences, Universitat Ramon Llull, Blanquerna, 08022 Barcelona, Spain

**Keywords:** physical activity, mobile health, smartphone, exercise, review, telemedicine, health promotion, health-related interventions

## Abstract

Unhealthy diet and physical inactivity—major risk factors for the main non-communicable diseases—can be addressed by mobile health applications. Using an evidence-based systematic review design, we analysed studies on mobile applications to foster physical activity to determine whether they met the objective of increasing adults’ physical activity. A bibliographic search was conducted in October 2020 using PubMed, Cochrane Library Plus, Biomed Central, Psychology Database, and SpringerLink, retrieving 191 articles. After titles and abstracts were reviewed, 149 articles were excluded, leaving 42 articles for a full-text review, of which 14 met the inclusion criteria. Despite differences in study duration, design, and variables, 13 of the 14 studies reported that applications were effective in increasing physical activity and healthy habits as dietary behaviour. However, further longer-term studies with larger samples are needed to confirm the effectiveness of mobile health applications in increasing physical activity.

## 1. Introduction

The main risk factors for the most important non-communicable diseases (cardiovascular conditions, type 2 diabetes, and certain types of cancer) are unhealthy eating habits and physical inactivity, which both greatly contribute to the global burden of morbidity, mortality, and disability [1]. Estimates for Europe indicate that more than a third of adults are not sufficiently active. The WHO European Region study entitled “Integrating Diet, Physical Activity and Weight Management Services into Primary Care” [2] confirmed the high European incidence of non-communicable diseases, accounting for 77% of the disease burden and nearly 86% of premature deaths. In 2016 the global prevalence of insufficient physical activity was 27.5%, and in high-income countries was more than double (36.8%) that of low-income countries (16.2%). Furthermore, in high-income countries, the incidence of insufficient physical activity has increased over time (31.6% in 2001) [3]. For the WHO European Region, two recommendations to tackle the epidemic of non-communicable diseases include a wide range of actions aimed at reducing risk factors, with primary care signalled to play “a fundamental role in the provision of services to promote healthy diets, involve people in the practice of physical activity, and help patients in weight management.”

The WHO defines physical activity as “any bodily movement produced by skeletal muscles that requires energy expenditure”, indicating that it is “a fundamental means of improving people’s physical and mental health” [1]. This further indicates that physical activity should not be confused with exercise, as the latter is “a subcategory of planned, structured, and repetitive physical activity”, whereas “physical activity includes exercise as well as other activities which involve bodily movement and are done as part of playing, working, active transportation, house chores, and recreational activities.” Participation in “150 min of moderate-intensity aerobic physical activity throughout the week or do at least 75 min of vigorous intensity aerobic physical activity throughout the week or an equivalent combination of moderate- and vigorous-intensity activity is estimated to reduce the risk of ischemic heart disease by approximately 30%, the risk of diabetes by 27% and the risk of breast and colon cancer by between 21 and 25%”. Mental health also benefits from physical activity, as stress, anxiety, and depression are reduced, and the effects of senile dementia or Alzheimer disease can be delayed [4].

A 2014 study entitled “Nutrition: The Impact of Smartphone Apps on the Nutrition Industry” [5] estimated that the global smartphone market would grow in 2017 to 3.45 billion users, and predicted an upsurge in applications (apps) specialized in nutrition, health, and fitness. A 2019 Spanish report on mobile use in Spain and worldwide indicates that 31.3 million people in Spain are smartphone users and estimates that 67% of all internet connections in the world are made from a smartphone. According to the same report, in 2018, 194 billion apps were downloaded worldwide (9% more than the previous year), 21.9% of smartphone users possessed a smartwatch, and lifestyle applications were the seventh most downloaded apps for iOS, Apple Inc., Cupertino, CA, USA [6].

Mobile apps can be effective in improving health outcomes and may come to be considered a means to developing cost-effective and scalable interventions. However, for interventions to effectively increase physical activity they need to motivate people to undertake behavioural change, offer realistic goals that can be combined with primary care guidance, and provide regular feedback on activity levels [7].

The use of mobile apps aimed at promoting physical activity linked to a healthy diet could further health education and help reduce non-communicable disease rates or, at the very least, enable monitoring and control of these diseases. Numerous reviews have already been carried out to determine the efficacy of health apps for weight reduction [8,9]. However, more interventions are necessary as well as subsequent reviews of apps that, either specifically or within a larger study, monitor physical activity to determine whether this increases/is maintained over time in response to appropriate motivation, given the risk that physical activity levels may taper off once an intervention ends [10,11].

The objective of this study, therefore, was to analyse the effectiveness of interventions based on mobile apps aimed at increasing physical activity.

## 2. Materials and Methods

We performed a systematic review of randomized clinical trials (RCTs) evaluating app efficacy in increasing physical activity. Electronic databases (PubMed, Cochrane Library Plus, Biomed Central, Psychology Database, and SpringerLink) were searched for articles published up to 23 October 2020. The following keywords and MeSH terms were used for the search: physical activity, physical fitness, cell phone, mobile app, sport, exercise, and smartphone. Boolean operators (AND, OR) were used to expand, exclude, or join keywords in the search. The search only considered RCTs conducted in adult humans whose results reflected changes in physical activity. The review is not restricted to comparing no app use groups and app use groups. The initial search, limited to publications in English and Spanish, was expanded by using the snowball technique to identify relevant articles in the references of retrieved articles [12]. The AMSTAR [13] and PRISMA [14] checklists were used to ensure the quality of the review. The risk of bias was assessed using Consolidated Standards of Reporting Trials (CONSORT) checklist [15] and disagreement regarding bias and the interpretation of results was resolved by consensus discussions.

### Study Selection

**Figure 1:**
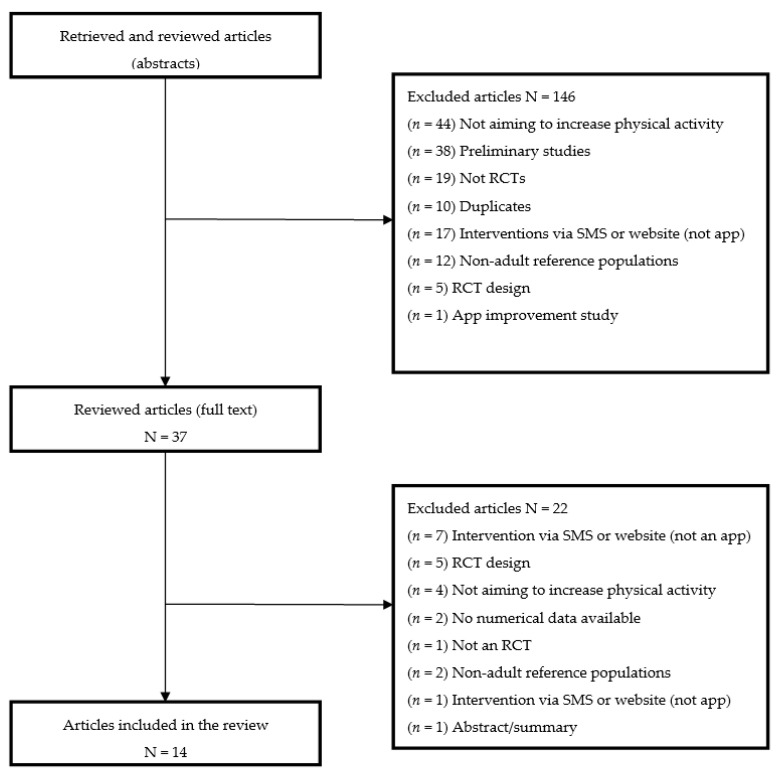
Search results and selection process.

A total of 191 studies were identified, whose titles and abstracts were reviewed against the inclusion and exclusion criteria (Table 1). After the review of titles and abstracts, 149 articles were excluded, 42 underwent a more detailed review consisting of reading the full text, and 14 articles were considered to meet the inclusion criteria. The main reasons for excluding articles were that the studies were not RCTs, only described study design, had reference populations that were non-adult, were missing data on physical activity variations, referred to improvements to apps, and were exclusively based on a website or SMS delivery (Figure 1). Studies in which participants might have some difficulty performing physical activity were not excluded if the objective of the study was aimed at improving it. To ensure the maximum reliability of the information collected from each article, the analysis was performed by two independent researchers [16]

We used a standardized form for extraction of the following data: study details, population and sample details, objectives, study type, study duration, intervention methodology, measures used, and results summary. Four authors reviewed the 14 articles independently and discrepancies were resolved by discussion with the lead author as necessary. All data were analysed both qualitatively and quantitatively.

## 3. Results

### 3.1. Population and Sample

The 14 selected studies (see Table 2 for details) were carried out in the USA (six studies [17,18,19,20,21,22]), Australia (two studies [23,24]), UK (one study [25]), Sweden (one study [26]), Israel (one study [27]), Pakistan (one study [28]), Denmark (one study [29]), and Belgium and Israel simultaneously (one study [30]). Study participant numbers ranged from 40 to 301 and nine of the 14 studies had sample sizes less than 100. Moreover, ten studies included participants of both sexes, although in seven of them women outnumbered men, three studies exclusively included men [23,24,25], and one study exclusively included women [27]. One study each was aimed at people with technical experience interested in healthy living [23], and at people without technical experience [19]. Participants were aged 18 to 69 years. Mean age reported was between 20.63 and 66 years. The body mass index (BMI, in kg/m^2^) of the individuals varied considerably (from 25.5 to 34.6), three studies did not report BMI [18,25,30], and two studies reported the percentage of overweight and obese participants by group [21,24]. Finally, only two studies provided information on abdominal circumference in women and men [17,27].

In the recruitment of participants, frequently taken into account was the level of physical activity (little, moderate or intense) or the need for no additional conditions limiting exercise capacity or non-participation in any other programme (e.g., for weight loss). No studies recruited athletes or physically very active persons, while two studies specifically recruited people with low levels of physical activity [19,20]. Patients with previously diagnosed pathologies were exclusively recruited in seven studies: obesity [17,22], type 2 diabetes [29], colon cancer [21], heart conditions [20], myocardial infarction [26], and Parkinson disease [30].

### 3.2. Interventions

In six of the studies [17,22,23,24,27,28], the goal of increased physical activity was part of a programme to change habits or lifestyle that also covered eating habits and weight loss. In the remaining eight studies, the main objective was to increase physical activity, although other secondary objectives may have been included, such as assessing quality of life, anxiety levels, or intervention adherence.

Intervention duration was six months or longer in five studies [17,21,22,24,26], two of which included follow-up [21,22], while duration was three months or less in the remaining nine studies.

All the studies required a mobile phone to be able to use the app. In some studies, participants were specifically required to have a smartphone or internet access [17,20,24,26,27,28,29], while a monitoring device was provided in other studies [18,19,21,22,23,25,30].

Of the 14 studies, nine were based on automatic recording of physical activity data (via pedometers and accelerometers), while the remaining five studies required users to manually enter physical activity data in the app [17,18,24,26,27].

Of the 14 studies, six included an intervention via app combined with SMS delivery, either as reminders or as reinforcement [17,18,20,26,27,28], while social interaction was included in the 8 remaining studies [19,21,22,23,24,25,29,30]. With the exception of studies 18 and 24 that included economic incentives linked to the increase in PA, the rest of the studies were based on socio-cognitive or behavioral theories for changing habits, including some kind of feedback (either individual or collective) as a motivational component [17,19,20,23,24,25,30], and offered educational information (face-to-face or through app educational modules) [17,21,23,24,26,27].

### 3.3. Measurements and Results

All 14 studies measured variation in physical activity from the beginning to the end of the intervention (depending on their duration, some studies included intermediate measures). Physical activity was measured in hours or minutes per day or week, number of sessions, and/or number of daily steps. Data was obtained automatically via an accelerometer or pedometer in 10 studies, or was self-recorded or obtained from the Global Physical Activity Questionnaire (GPAQ) or International Physical Activity Questionnaire (IPAQ) in the remaining four studies. Sedentary lifestyle details were explicitly recorded in one study [19], while perceived barriers were considered in three studies [17,25,29].

Other measures considered were weight loss [17,22,26], quality of diet and dietary habits [23,24,27], BMI [17], wellbeing [23], lifestyle changes and mental health (distress, anxiety, depression, etc.) [21], and health literacy [24]. Frequency of app use, app usability, and participant satisfaction were other measures common to several studies [18,20,23,24,27,29].

In the studies aimed at patients with specific pathologies, variables typical of the condition of interest were also measured. For instance, in their obesity study, Allen et al. [17] measured abdominal circumference. In their study focused on myocardial infarction, Johnson et al. [26] measured adherence to ticagrelor as the primary outcome, and also changes in cardiovascular risk factors, quality of life, and patient satisfaction with the intervention. In relation to Parkinson disease, Ginis et al. [30] focused on gait speed (not only comfortable speed, but also double-task speed), secondary gait, freezing-of-gait severity, balance, and Parkinsonian-related and other cognitive evaluations.

### 3.4. Analysis

Despite differences in duration, design, and variables, 13 of the 14 included studies reported an increase in physical activity. The only exception was the pilot study by Allen et al. [17], whose results were not statistically significant, probably due to the small sample size and less than robust retention strategies. Although the increases in physical activity were moderate, the studies that included other variables also highlighted improvements in diet [23,24], weight loss [27,28], or adherence to treatment [26]. However, in some cases it was reported that increased physical activity [21] or weight loss [22] were not maintained after the intervention ended at nine or 18 months, respectively. Naimark et al. [27] also demonstrated the positive impact of an app, although they indicated that long-term studies would be necessary to reach more definitive conclusions.

Some characteristics and components of the apps associated with effectiveness in increasing PA seems to be related with setting goals or receiving feedback, rewards, or educational information led to improved motivation and results [18,19,24,25,26,27]. Other findings were that receiving motivational messages was perceived as a positive factor [18], apps adapted to people with little experience of technology were well received [18], people with less technical knowledge had more problems adhering to technology-based interventions [30], and men were possibly less likely to respond to reminders than women [29].

Regarding acceptance and satisfaction with the interventions, study participants valued very positively personal or group sessions and the use of mobile apps. Users who did not use an app considered that a data recording device would have enhanced their motivation [17]. Although the participants stated that they remained motivated and eager to continue with the intervention [20,29,30], or intended to continue using the app [27], in several studies (especially the longer ones) a decrease was observed in the use of the apps over time [18,21,22,24,28].

## 4. Discussion

Our review of different studies of mobile apps used to promote physical activity suggests that such interventions are well accepted and can facilitate success in achieving moderate or intense physical activity levels. Although the analysed studies are generally of short duration and the increases in physical activity are modest, the accessibility and flexibility offered by the applications could be useful to increase the number of people who adhere to the recommended levels of PA and thus reduce the risk of non-communicable diseases such as coronary heart disease, obesity or type 2 diabetes mellitus [31]. Although the increase in physical activity seems to be greater in those applications aimed only at increasing PA, it is important to consider that the studies that also include other healthy behaviors have positive results as well [23,24,25,26,27,28]. Therefore, it could be interesting to work in conjunction to promote healthy lifestyles and thus have a positive impact on reducing costs for the health system.

Around a quarter of the participants in the study by Allen et al. [17] considered the use of a smartphone app the most useful aspect of the programme, while all the participants considered that a tracking device would enhance their motivation to increase physical activity levels. Likewise, several studies reported that users were interested in continuing with the programme and using the app, and would recommend it to friends [27,29,30]. This would suggest that health apps may not only help users maintain a healthy lifestyle but that their use could be transmitted at the social level.

Regarding the association between greater use and goal achievement, Naimark et al. [27] showed that heavier device users were more oriented towards a healthy lifestyle and tended to obtain better results (measured as the number of steps). Adherence to app-based treatment is an important issue in greater frequency of use [32], while it has been suggested than an automatic step-counting system could improve the acceptance of strategies to increase physical activity [17]. Message delivery [20] and individual and social feedback [18] were reported to be key factors in the success of some interventions.

Our review has some limitations that need to be taken into account in future studies. The number of participants in the different studies, as well as study duration, would suggest a need to carry out longer-term studies with larger samples to determine if interventions would continue to retain user interest and whether they would be sustained over the long-term [17,19,27,30]. Given that several studies link sedentary lifestyles with mortality, King et al. [19], for instance, strongly recommended expanding RCTs.

Another limitation was that only some of the included studies included post-intervention follow-up [21,22,30].

Regarding sampling, a possible limitation to interpreting results is that groups need to be balanced in number and sex [27]. The study by Valentiner et al. [29] suggests that men may show less involvement than women in responding to messages. Other studies confirm the greater predisposition of women to actively participate in programmes compared to men [33,34]. King et al. [19] suggested, in relation to behavioural interventions like that of their study, that subgroup analyses were necessary to identify tailored interventions for particular individuals and circumstances, as this would “improve the personalization of e-health interventions and optimize success”. Harries et al. [25], for instance, whose study recruited only men, considered that women’s clothing might mean they would not always carry their smartphone with them. This would suggest that, in designing effective intervention programmes, it is essential to understand psychological determinants underlying self-management of a change in habits [27].

For interventions involving automatic data monitoring devices, the choice of accelerometer or pedometer is important in terms of ensuring reliable data collection, as initial testing is often necessary to calibrate the sensitivity of devices [10]. According to King et al. [19], the fact of needing to have the device constantly to hand could represent a limitation, with the same authors suggesting that the challenge is to develop monitoring devices capable of accurately capturing not only physical activity, but also sedentary and sleep habits 24 h a day. Continuous monitoring that provides immediate feedback is a motivator for change, as is manual recording (once it becomes a habit) and dynamic information on progress, as such motivations improve the efficiency of a programme [25,35].

The ethical issues regarding the collection of personal data need to be taken into account. Beyond the need to respect data protection legislation, the user must also consent to provide data. In the case of the Spanish population, very concerned regarding the presence and use of their data online, 47% admit that they did not fully review permissions for installed apps, and 21% do not do so for newly installed apps [6].

Martin et al. [20] considers that not having a professional to motivate participants through personal interviews is a possible limitation, despite the fact that their study obtained good results without such a professional. Spring et al. [22], however, demonstrated that a coach interacting with the user during the programme improved effectiveness for the intervention group compared to the control group.

## 5. Conclusions

People are increasingly using smartphones routinely in more areas of their personal and professional lives. In the health field, apps are becoming an increasingly used means not only to speed up medical consultations, but also to promote education in healthy lifestyles, proper diet, and physical activity.

Mobile apps aimed at increasing physical activity can be effective in helping people acquire healthy habits. Our review of 14 articles, despite differences in study duration, design and variables, and despite the single exception of the pilot study by Allen et al. [17], would broadly suggest a clear trend – that the use of apps increases physical activity. Many of those studies also suggest that users would be willing to continue using the apps and would even recommend them, thus expanding the scope of health interventions.

However, the success and effectiveness of health interventions and apps relies on their adaptation to the target, attractiveness, ease of use, and adherence. Personalized interventions and apps are key to achieving success with manageable goals adapted to individuals, their circumstances, and their progress.

Because motivation is essential to making sustainable changes in behaviours and lifestyles that persist beyond the end of an intervention, attention must be paid to the success factors as highlighted by many of the studies that analysed support and reminders, health education, and feedback on goals and results. Social interactions, whether with other people using the app or with a health professional, also seem to play a key role in increasing the effectiveness and sustainability of interventions. Interpersonal contact would therefore seem to be an important issue to take into account when developing mobile health apps.

A crucial aspect of developing and implementing mobile apps is that they must comply with ethical and health criteria and with data protection legislation, and that they must obtain the user’s consent to the use of their data. Likewise, users would need to thoroughly understand the app to ensure that it is used efficiently and safely.

Further longer-term studies with larger samples are needed to confirm the effectiveness of apps in increasing and maintaining physical activity levels over the long run. Going beyond usability and retention analyses, research is also needed concerning ethical and privacy aspects with a view to conducting exhaustive analyses of interventions aimed at enhancing the efficacy and safety of the growing number of mobile health apps.

## Figures and Tables

**Table 1:** Inclusion and exclusion criteria.

Inclusion Criteria
RCT: use of mobile apps that promote exercise and/or physical activity. Language: English or Spanish. Study population: adults (18 years and older).
**Exclusion Criteria**
Opinion articles, editorials, and commentary interpreting published results. Several articles published on the same study by the same authors: the latest study was selected, and all other studies were excluded. Informative material. Interventions based exclusively on websites or SMS use (not on app use).

**Table 2:** Details of the 14 reviewed articles.

Study	Population and Sample	Objective(s)	Type & Duration	Intervention	Measures	Results	Conclusions
Allen [17]	*N* = 68 Age: 21–65 y BMI: 28–42 USA Women: 78% Means: Age: 44.9 y Weight: 97.3 kg BMI: 34.3	To evaluate feasibility, acceptability and preliminary efficacy of smartphone-based behavioural interventions	Pilot RCT 6 mo	App-based behavioural interventions plus professional guidance Goals: 5% weight loss and 150 min MVPA Groups: **Group IC (*N* = 18)** -Intensive counselling **Group IC+SP (*N* = 16)** -Intensive counselling -Smartphone intervention **Group LIC+SP (*N* = 17)** -Less intensive counselling -Smartphone intervention **Group SP (*N* = 17)** -Smartphone intervention	**Start and 6 mo** -Weight, height, BMI -PA (7D-PAR) -Estimated energy expenditure -Food intake over 3 d -Diet and PA record monitoring. -Intervention satisfaction (in-depth interviews)	**Change at 6 mo** **PA** (mean h/wk) -(IC): −1.4 -(IC+SP): −2.0 -(LIC+SP): −3.6 -(SP): 0.19 **BMI** -(IC): −0.8 -(IC+SP): 1.8 -(LIC+SP): −1.1 -(SP): −0.7 **Weight loss** ≥5%: 64% (IC+SP); 40% (LIC+SP) ≤5%: 25; (IC); 20% (SP) **Other** -Improved diet **Retention:** 59–69%	-No statistically significant differences between the 4 groups -Satisfaction and improvements: possibility of automatic PA and weight recording -No dropout differences by sex or ethnicity
Ashton [23]	*N* = 50 Age: 18–25 y Men Australia Means: Age: 22.1 y BMI: 25.5 Steps/d: 6994.4	To evaluate programme (Heyman) viability and impact on PA levels (steps/d and MVPA), diet, subjective wellbeing and other measures	Pilot RCT 3 mo	Intervention based on behavioural theories on changing habits. Groups: **C: Control (*N* = 24)** Life as normal for 3 mo on intervention waiting list **I: Intervention (*N* = 26)** -Website with resources -Jawbone wearable PA tracker with associated app for goal setting and health behaviour self-monitoring -Group/individual face-to-face sessions -Private Facebook discussion group -Gymstick™ resistance band. -Portion planner disc™	**Start and 3 mo** -Change in steps/d: pedometer -Diet quality (score): AES-FFQ -Changes in lifestyle, psychological, anthropometric and physiological measures	**Change at 3 mo** **PA** Pedometer (steps/d) -C: 575.4 -I: 1588.2 MVPA (min/wk) -C: 26.1 -I: 154.1 **Diet quality (score)** -C: 2.3 -I: 5.9 **Wellbeing (total score)** -C: 0.5 -I: 0.9 **Other** Improved diet, weight loss, other **Use and acceptability** Reasonable levels for most programme components **Retention**: 94%	-No significant between-group differences for steps/d, diet quality or wellbeing -Viability of programme demonstrated for a subsequent RCT -RCT not designed to detect between-group differences
Duncan [24]	*N* = 301 Men Age: 35–54 y Australia	To assess effectiveness of technology-based (IT) compared to paper-based (IP) interventions in improving PA, eating behaviours and health literacy	RCT 9 mo	Social cognitive theory/self-regulation theory. ManUP intervention based on challenges (6 PA+1 diet) adapted to starting level (light, moderate, intense) Groups: **IP (*N* = 96)** -Hardcopy educational PA and healthy diet materials-Progress self-monitoring using recording forms -No information provided on other participants -Hardcopies not collected -> no information obtained on challenges or self-control **IT (*N* = 205)** -Material as for IP group -Automatic feedback on progress and goals -Possibility of social (website) interaction with other participants	Online surveys at 0, 3, 9 mo **PA** AAS (total min. PA + number of PA sessions/wk) **Diet** Adapted Australian population survey (strong psychometric properties) **Health literacy** PA and diet surveys **Satisfaction** Likert scale	**PA (min/wk)** -IT vs IP = 1.03 3 mo vs 0 = 1.45; 9 mo vs 0 = 1.55 **PA (sessions/wk)** -IT vs IP = 0.97 3 mo vs 0 = 1.61; 9 mo vs 0 = 1.51 **Diet (score)** -IT vs IP = 1.02 -3 mo vs 0 = 1.07; 9 mo vs 0 = 1.10 **Health literacy** -Significantly more IT than IP participants considered MVPA of 20 min/d × 3 d/wk to be essential for health **Retention:** 49.2% (lower in IT group, 46.8%)	-ManUp effective in improving PA and diet, but no significant differences between interventions.
Fanning [18]	*N* = 116 Age: 30–54 y Sedentary participants (<30 min MVPA × 2/wk) USA Means: Age: 41.38 y Women 80%	To determine individual and combined impact of a self-monitoring app and 2 theoretical modules (goals and rewards) on moderate/intense PA, psychosocial outcomes and app use	RCT 12 wk	Interventions based on social cognitive theory plus S.M.A.R.T. goals Groups: **A (*N* = 29)** -Basic app -Goals module -Rewards system (points) **B (*N* = 31)** -Basic app -Goals module **C (*N* = 26)** -Basic app -Rewards system (points) **D (*N* = 30)** -Basic app	**PA** -Actigraph accelerometer × 7 d (wk 1 and wk 12) -MVPA (>1952 counts/min) **OTHER** **Self-efficacy** -Modified BARSE -Modified EXSE -LSE **Perceived barriers** -Perceived barriers scale **Expected results** -MOEES **Goals** EGS **Use and usability** -Access recorded to apps -Open questions on acceptability -Ease/difficulty associated with use of each module (5-point Likert scale)	**PA**- from 34.88 to 46.77 min in MVPA - increase 11.90 min/d PA in conditions (d = 0.70) −5.94 extra min/d PA for rewards module **Psychosocial variables** -Less self-efficacy in overcoming barriers for no-points module (d = −0.39) **PA self-efficacy** 3-way time-points-goals interaction was significant (*p* = 0.01) **Lifestyle self-efficacy** Only time-point interaction was significant (*p* = 0.03). **-Goal setting** -06:59 more units in perceived ability to set goals for the intervention (d = 0.82). -Better perceived ability to set goals with the points system (d = 0.99) **Expected results** Slight-moderate increase for points system (d = 12:28), decrease for no-points system **Self-assessment** (*p* = 0.07) Slight-moderate increase for points system (d = 12:25), decrease for no-points system **Retention:** 88%	-Individuals in all conditions improved daily PA -The rewards module was effective in promoting PA change -Positive evaluation of motivational SMS and request for more such SMS
Ginis [30]	*N* = 40 Age: N/K Participants with Parkinson on stable medication, able to walk 10 min non-stop MoCA score ≥24 Belgium & Israel	To determine feasibility and effectiveness of real-time feedback on gait performance (CuPiD) compared to conventional gait training in the home setting	RCT6 wk + 4 wk follow-up	Groups: **I: CuPiD (*N* = 22)** -Weekly home visits -Gait training 3 × 30 min/wk -Phone with ABF-Gait app: positive/corrective comments on the fly **C: Active control (*N* = 18)** -Weekly home visits -Gait training 3 × 30 min/wk -Personalized on-the-fly advice -No CuPiD	**Gait speed (primary results)** -Walk 1 min on treadmill -Usual conditions: comfortable dual-task (DT) speed while reciting as many words as possible starting with a pre-specified letter **Secondary gait and balance** -2MWT -Mini-BESTest -FSST -FES-I -PASE **FOG severity** NFOG-Q and Ziegler protocol **Cognitive evaluations** CTT and VF when sitting and walking	**Gait speed (M/s)** **Start** Significant improvement at both speeds for (I) and (C): -(I) 9.0% (comfortable) and 13.5% (DT) -(C) 5.2% (comfortable) and 5.8% (DT) **Stamina and physical capacity** **2MWT** **Start** Gait improvements were also noticeable for the 2MWT **PASE (0–400)** (I): balance significantly improved (Mini-BESTest) in post-test (from 24.8 to 26.1, SD = ~5) (I): QoL maintained (SF-36) at follow-up (C): QoL reduced (from 50.4 to 48.3, SD = ~16) at follow-up Other between-group differences were not significant	-CuPiD was feasible, well accepted and effective in promoting gait training, with participants improving in equal measure -The impact of (C) can be interpreted as small, while that of (I) can be considered clinically moderate and comparable to similar studies -Balance was improved more with(I) than with conventional training for Parkinson
Harries [25]	*N* = 152 Age: 22–40 y Men UK Means: Sedentary work 50%. Regular sports 59% Motorized transport 63%	To determine impact of feedback on number of steps	RCT 8 wk	Groups: **C: Control (*N* = 49)** No feedback or access to interactive app functions **II: Individual feedback (*N* = 53)** Personal feedback on steps **IS: Social feedback (*N* = 50)** Individual compared to group feedback on steps	**Steps/d** App measurement of steps/d **Other** Attitudes to PA and perceived barriers (start and end surveys)	**PA** Mean steps/d recorded: (C) = 2822 (II) = 3842 (IS) = 3984 Compared: (II) vs (C) +60% (IS) vs (C) +67% **Other** -Any form of feedback (II) and (IS) explained 7.7% of inter-subject variability in step count (F = 6.626, *p* < 0.0005) -Differences between the 2 intervention groups were not statistically significant **Retention:** 92%	-Apps to count steps can increase PA in young men -Feedback increased PA, but there were no significant differences between the 2 feedback groups
Johnston [26]	*N* = 166 Age: >18 y Patients with myocardial infarction receiving ticagrelor Sweden Means: Age: 58 y Men: 81% BMI: 29 Diabetes: 13% Smokers: 21%	To evaluate an app aimed at improving treatment adherence and lifestyle in patients with myocardial infarction	Multi-centre RCT 6 mo	Groups: **I: Intervention (*N* = 86)** -Interactive patient support app -Missed dose: SMS the next day + educational message -Prevention education modules (referenced medical information) and personalized message (status, progress) -Extensive treatment adherence module -Exercise -Weight -Smoking -Possibility of recording blood pressure, cholesterol and glucose **C: Control (*N* = 80)** -Simplified app -Missed dose SMS the next day	**Primary measure** Adherence to ticagrelor **Secondary measures** -Changes in cardiovascular risk factors (BMI, PA, smoking) -QoL -Satisfaction **Scales and surveys** -EQ-5D VAS (visits 1, 2, 3) -PA surveys (visits 1, 2, 3) -MARS-5 (visits 2, 3) -SUS (visits 2, 3) Support app also evaluated for active group	**PA** **PA sessions/wk (SD)** (I) = +1.5 (C) = +1.0 **PA m/wk (SD)** (I) = +1.5 (C) = +65.0 **PA > 150 m/wk** (I) = +33.8% (C) = +21.1% **Change in QoL** (EQ-5D VAS) 14.7 vs 8.4 (*p* = 0.059) Positive trend for (I) with respect to (C) but not statistically significant **Satisfaction** Significantly higher in (I) vs (C): SUS 87.3 vs 78.1 (*p* = 0.001)	-PA increased in (I) compared to (C) -Users would recommend use of app to others
King [19]	*N* = 89 Age: >45 y No smartphone experiences MVPA <60 min/wk Seated >10 h/d USA Means: Age: 60 y Women: 75.3% BMI: 28.8	To evaluate 3 personalized PA apps based on conceptually different motivational frameworks in comparison to a commercial control app	RCT 8 wk	Groups: **C: Control (*N* = 24)** -Diet control to monitor daily eating behaviour **Custom apps sharing basic functions:** **I-1: Analytical app (*N* = 21)** -Emphasis on personalized and quantitative goals, behavioural feedback, tips to promote behavioural change and problem-solving strategies **I-2: Social app (I-2) (*N* = 22)** -Emphasis on social support for behavioural change, normative social feedback, behaviour modelling and group collaboration and competition **I-3: Affective app (*N* = 22)** -Reinforcement programming, reasons and motivation for connection and gamification	**Daily PA and sedentary behaviour** -Smartphone accelerometer -Valid data (h) or no more than 60 consecutive 0 values (non-wear time) -Valid day: minimum 10 valid h/d -MVPA (>301 counts/min) -Sedentarism (<56 counts/min) **Daily self-report measures** -EMA -Brisk walking min/d -Sitting time, h	**Moderate/intense PA** Differences between groups *p* = 0.04-0.005 (I-2) vs (C): d = 01:05, CI = 0.44, 1.67 (I-2) vs (I-3): d = 0.89, CI = 0.27, 1.5 (I-2) vs (I-1): d = 0.89, CI = 0.27, 1.51 **Sedentarism** Differences between groups *p* = 0.02–0.001 (I-2) vs (C): d = 1.10, CI = 0.48, 1.72 (I-2) vs (I-3): d = 0.94, CI = 0.32, 1.56 (I-2) vs (I-1): d = 1.24, CI = 0.59, 1.89 **Seated time** Differences between groups *p* < 0.001 (I-2) vs (C): d = 1.59; CI = 0.92, 2.25 (I-2) vs (I-1): d = 1.89, CI = 1.17, 2.61 (I-3) vs (C): d = 1.19, CI = 0.56, 1.81 (I-3) vs (I-1): d = 1.41, CI = 0.74, 02.07 91.3% of social app users used the message board (total: 775 SMS). **Retention:** I-1 (95%), I-2 (100%), I-3 (92%), C (89%)	-Social app users significantly increased MVPA (weekly accelerometer) relative to the other 3 groups -Social app users overall had significantly greater accelerometer-derived sedentarism -Social and affective app users reported less time seated than users in the other 2 groups -Satisfaction was high among users -No significant between-group differences in app use
Martin [20]	*N* = 48 Age: 18–69 y Cardiology outpatients MVPA for ≥30 min/d for less 3 day/week USA Means: Age: 58 Sex: Men 54% BMI: 31 Diabetes: 23% Cardiopathy 29% Hypertension: 50% Steps/d: 9670	To assess if a fully automated mHealth intervention with tracking and texting increased PA	Pilot RCT 5 wk	Fitbug Orb (app with accelerometer linked to intelligent SMS system) Goal 10,000 steps/d Groups/phases: **PHASE I (wk 2–3)** **I: Nonblind (*N* = 32)** -Tracking access via website or app: -steps/d- activity time -aerobic activity time -previous data history **C: Blind (*N* = 16)** -No access to tracking **PHASE II (wk 4–5)** **I-1: Tracking and SMS (*N* = 16)** -Access to tracking -Personalized SMS in doctor’s name (x 3 per d) -Positive reinforcement SMS -Reinforcement SMS **I-2: Tracking (*N* = 16)** -Access to tracking/no SMS **C: Blind (*N* = 16)** -No tracking access	**Primary result** -Mean change in accelerometer-counted step/d from baseline to phases I and II -Achievement of goal of 10,000 steps/d **Secondary results** -Change in PA/d -Change in aerobic time (>10 min continuous walking with no pause >1 min) -Satisfaction (end-of-trial online survey, with qualitative and quantitative elements).	**PHASE I** **Change in steps/d** No significant change/difference between groups **Activity min/d** No significant change/difference between groups **Aerobics min/d** Smaller significant decrease in time limit in (I) (8 min difference, 95% CI: 0–16, *p* = 0.05) **PHASE II** **Change in steps/d** 37% absolute increase/84% relative increase in (I-1) over other groups at 10,000 steps/d (*p* = 0.02) **Activity min/d** Increase in (I-1) by 21 min/d (+23%) **Aerobics min/d** Statistically very significant increase in (I-1) by 13 min/d (+160% relative to other groups) **Satisfaction** -PA tracking: mean 4.0 out of 5.0 -SMS: mean 3.8 out of 5.0	−48% of participants achieved 10,000 steps/d at the study start. -PA trajectories were different for the 3 groups: For (C), but not for (I-1) or (I-2), there was a progressive downward trend over time, while for (I-1) the clear upward trend was due to SMS -PA increased with automatic intervention with but not without SMS -> increase depended on the SMS component
Mayer [21]	*N* = 284 Age: ≥21 y Colon cancer (stage I-III, treatment completed) PA level <150 min/wk Absence of other cancers (except skin cancer) USA Means: Age: 58 y Men: 48.5% Caucasian: 89% Obesity: 69.5%	To assess Survivor-CHESS app impact on PA in colon cancer survivors and explore Survivor-CHESS impact on QoL and anxiety	RCT 6 mo + 3 mo maintenance	Goal: PA 150 m/wk Groups: **C: Control (*N* = 140)** -NCI booklet: *Facing Forward: Life After Cancer Treatment* -NCCS Cancer Survival Toolbox -Pedometer. **I: Intervention (*N* = 144)** -Material as delivered to (C) -Smartphone with SurvivorCHESS app (voice/data service) with: -skills development (My Tracker/Be Mobile). -support services (My Friends). -information services and tools (My Cancer Care) Note: After 6 mo, certified trainer available to users through app	**Start** -Demographic and medical data on cancer -BMI -Comorbidity conditions (OARS) **App (group (I) only)** -Number of session started -Pages viewed -SMS content -Internet use convenience (5-point Likert scale: 0–4, min-max). **PA** -GPAQ: during 1 wk, mean for exercise type (intense, moderate, light) >15 min -Total min: weekly frequency of MVPA	**PA** (I) 19.4–60.0 min (MPA) (C) 15.5–40.3 min (MPA) (*) Non-significant intervention effect (F(1, 221) = 2.404, *p* = 0.122) **9 mo** -Intervention effect for the same outcome at 9 mo controlling for outcome at 6 mo was not statistically significant (F(1, 202) = 0.722, *p* = 0.396) -No significant between-group differences for intervention effect sustainability at 9 mo **Dropout** (6 mo): (C) 28.1% (I) 18.2%	-Greater increase in MVPA in (I) relative to (C) -PA increased over time in both groups with no significant between-group differences -Patients with higher BMI and more comorbidities were less likely to increase PA
Memon [28]	*N* = 56 Age: 18–25 y Women BMI > 25 Pakistan	To evaluate PA increase and weight loss in university students using financial incentives and a smartphone app	RCT 5 wk	Groups: **I: Intervention (*N* = 28)** -Increasing financial incentives for steps/d -ProtoGeoO app **C: Control (*N* = 28)** -No financial incentive -ProtoGeoO app	**PA** -Steps/d measured by app **Demographics** -Various questionnaires -BMI **Secondary variables** -Body image perceptions -Anxiety -Weight control strategies Measurements based on various questionnaires	**PA** (C) = 47314.36 steps vs (I) = 57799.61 steps, *p* > 0.05 **Weight loss** (C) 68.67 kg (start) to 67.96 kg (end), *p* = 0.004 (I) 72.13 (start) to 70.97 kg (end), *p* < 0.001 **Retention:** 100%	-Notable weight loss in both groups after 5 wk. -No significant difference between the 2 groups in weight loss and steps increase -Significant drop in app use over time
Naimark [27]	*N* = 85 Age: >18 y Technical experience Healthy living interest Israel Means: Age: 47.9 y Women: 64% BMI: 25.8	To compare adherence to a healthy lifestyle between an app group receiving educational information and a group receiving only educational information	RCT 14 wk	Groups: **I: Intervention (*N* = 56)** -Introductory session on healthy lifestyles, weight change, nutrition, PA -Access to eBalance app without face-to-face support -Diet and PA control tools that also educate on health **C: Control (*N* = 29)** -Introductory session on healthy lifestyles, weight change, nutrition, PA -Life as normal	**Start and wk 14** -Weight -Waist circumference -Evaluation (online surveys) on nutritional knowledge, diet quality and PA (28 items on type, frequency, duration/wk) **Wk 14** -App usability (frequency and convenience) -Satisfaction questionnaire	**PA** **Low activity <150 min** -Start: (C) 45% (I) 28% -Wk 14: (C) 55% (I) 17.3% **Recommended 150–300 min**-Start: (C) 30% (I) 36.5% -Wk 14: (C) 25% (I) 32.7% **High activity >300 min** -Start: (C) 25% (I) 34.7% -Wk 14: (C) 20% (I) 50% **Mean PA change** (I) 63 min (SD 20.8) vs (C) −30 min (SD 27.5), **Mean weight and BMI change** (I) weight −1.44 kg (SD 0.4), BMI −0.48(C) weight −0.128 kg (SD 0.36), BMI −0.03 **Retention:** 86%	-App motivates users to significantly increase PA time/wk. More users increased PA to >150 min/wk. -Greater weight loss for (I) than (C) -Significant increase in nutritional knowledge in (I) -Frequent app use was significantly associated with greater success (*p* < 0.001) -Most users stated that the app helped them/they would recommend it to others
Spring [22]	*N* = 96 Age: 18–60 y BMI: 30–40 No weight loss >11.3 kg in previous 6 mo USA Means: Age: 39.3 y Women: 84.4% Weight: 94.8 kg BMI: 34.6	To determine the impact of 3 weight loss interventions with/without training and mobile technology	RCT 6 mo + 12 mo follow-up	ENGAGED intervention: technical and social weight control measures Groups: **SELF: self-guided (*N* = 32)** -Calorie counting book -Hardcopy self-monitoring diaries (6 mo): diet, PA, weight **STND: standard (*N* = 32)** -As for SELF -8 group sessions -Coaching phone calls (1/wk first 8 wk, then 1/mo) **TECH: technical (*N* = 32)** -Smartphone -App -Shimmer accelerometer for 6 mo -8 group sessions -Coaching phone calls -SMS and social network At 3 and 6 mo, competition between groups with financial incentives	**Primary results** -Weight (start, 3 mo, 6 mo, 12 mo) -Significant weight loss (≥5%) -Goal for all groups: 7% weight loss (approx. 0.5–1 kg/wk) **Behavioural adherence (mo 1-6)** -Diet self-monitoring: % days with intake ≥1000 cal/d -PA self-monitoring: % days PA reported or detected -Goals (45–175 min/wk) **Fidelization** -Phone call/monitoring checklist: 2 × mo for mo 1–2, then 1 × mo from mo 3 (to intervention end)	**Weight loss** **6 mo** -Higher in TECH and STND than in SELF (25.7 kg [95%CI: 27.2–24.1] vs 22.7 kg [95%CI: 25.1–20.3], *p* < 0.05) **12 mo** -Loss ≥5% for 47% STND, 28% TECH and 25% SELF (non-significant differences) **Self-control adherence (6 mo)** -Self-control of diet, PA and weight (% day adherence) greater for TECH and STND than for SELF (*p* < 0.001) -Higher PA in TECH 56.8 (4.8) than in STND 30.5 (4.4) or SELF 9.8 (2.4) **Treatment fidelity** -Training time (1–6 min): greater for TECH (285.71 min [SD = 83.9]) than for STND (202.8 min [SD 89.4]): F(1, 61) = 14.39, *p* < 0.001 **Dropout (12 mo)** -Higher for SELF (25.0%) than for STND (12.5%) or TECH (3.1%)	-Self-control adherence to PA higher for TECH than for SELF and especially so than for STND -Weight loss was not significantly different in any group at 12 mo
Valentiner [29]	*N* = 37 Age: 30–80 y DM-II Denmark Means: Age: 66 y Women: 65% BMI: 28.5 Body fat: 37.9%	To investigate feasibility of IWT adherence using EMA and InterWalk in patients with DM-II	RCT 12 wk	Groups: **C: Control (*N* = 19)** -InterWalk for IWT (>90 min × ≥3 d/wk) **I: Intervention (*N* = 18)** -App use as for controls -Individual goals via interview -Automated survey each wk -Phone call on IWT barriers	**PA** -Adherence to IWT (total accumulated time during the intervention in InterWalk data) **Other secondary measures** (exploratory) -Usability of SMS -Self-reported PA -Satisfaction with trial participation -Quality of life (Short-Form Health) Survey (SF-12) -Anthropometric measurements	**PA** -I: 434 min overall more than C -I: 36 min/wk more than C -Goal achievement: 47% I and 11% C **Usability** -Women more participatory in the experimental group **Satisfaction and perceptions** −68% very satisfied −78% intended to continue using app after intervention end **Retention:** 100%	-The I combination is suitable for achieving IWT adherence -Men respond less to SMS than women

**Abbreviations:General:** BMI, body mass index (kg/m^2^); DM-II, diabetes mellitus 2; FOG, freezing of gait; IWT, interval walking training; M, metre; mHealth, mobile health; MPA, moderate physical activity; MVPA: moderate-to-vigorous physical activity; NCCS, National Coalition for Cancer Survivorship; NCI, National Cancer Institute; PA, physical activity; QoL, quality of life; RCT, randomized controlled trial; S.M.A.R.T. (goals), S-specific, M-measurable, A-attainable, R-realistic, T-time oriented. **Time:** d, day; h, hour; min, minute; mo, month; s, second; wk, week; y, year; ×, times. **Instruments:** 2MWT, Two-Minute Walk Test; 7D-PAR, Stanford 7-Day Physical Activity Recall; AAS, Active Australia Survey; AES-FFQ, Australian Eating Survey Food Frequency Questionnaire; BARSE, Barriers Self-Efficacy Scale; CTT, Color Trail Test; EGS, Exercise Goal-Setting Scale; EMA, Ecological Momentary Assessment; EQ-5D European Quality of Life-5 Dimensions; EXSE, Exercise Self-Efficacy Scale; FES-I, Falls Efficacy Scale International; FSST, Four Square Step Test; GPAQ, Global Physical Activity Questionnaire; LSE, Lifestyle Physical Activity Self-Efficacy Scale; MARS-5, Medication Adherence Report Scale; Mini-BESTest, Balance Evaluation Systems Test; MoCA, Montreal Cognitive Assessment; MOEES, Multidimensional Outcome Expectations for Exercise Scale; NFOG-Q, New Freezing-of-Gait Questionnaire; OARS, Older Americans Resources and Services Multidimensional Functional Assessment Questionnaire; PASE, Physical Activity Scale for the Elderly; SF-36, Short Form-36 Health Survey; SUS, System Usability Scale; VAS, Visual Analogue Scale; VF, Verbal Fluency Test.
